# EXPLORING PROPERTY TAX BURDENS AND AGING IN PLACE: A GEOSPATIAL INVESTIGATION IN PENNSYLVANIA

**DOI:** 10.1093/geroni/igad104.3331

**Published:** 2023-12-21

**Authors:** Jongwoong Kim

**Affiliations:** West Chester University, West Chester, Pennsylvania, United States

## Abstract

Utilizing Geographic Information System (GIS) techniques and a composite index, this project delves into the intricate geographic patterns of property tax burdens in Pennsylvania, with a specific focus on older residents contemplating aging in place amidst limited financial resources. The study scrutinizes various factors, including property tax rates, home values, income, demographics, and other housing-related variables, to comprehensively grasp their effects on the financial feasibility of aging in place. Pennsylvania displays notable disparities in property taxes, averaging approximately 1.5% of home value, across counties due to distinct assessment systems and varying assessed-to-market ratios (CLRs). Tax rates are also influenced and specified by smaller administrative and geographic entities, including municipalities and school districts. Despite alternatives (e.g., assisted living), aging in place remains the preferred choice for most older Americans. This accentuates the significance of assessing property tax affordability that facilitates potential aging in place, particularly for financially constrained but “healthy” seniors who may not qualify for tax rebates or local freezes. Moreover, escalating housing prices and assessed values further emphasize the urgency. Cartographic and tabular findings from the study unveil challenging situations for seniors in specific county clusters of Pennsylvania, encompassing not only “expensive” urban but also “typically affordable” rural settings. Additionally, areas with the highest tax rates are not always the most financially burdensome (and vice versa) with all other factors taken into account. This study offers practical insights that have the potential to inform adjustments in housing and tax policies aimed at benefiting older adults in Pennsylvania.

